# Tidal continuous cycling peritoneal dialysis in children

**DOI:** 10.1007/s00467-023-05898-x

**Published:** 2023-02-13

**Authors:** Lavjay Butani, Maha Haddad, Mark Joseph

**Affiliations:** 1 Division of Pediatric Nephrology, Department of Pediatrics, University of California, Davis, 2516 Stockton Blvd, Room 348, Sacramento, CA 95817 USA; 2 Division of Pediatric Nephrology, Department of Pediatrics, Oregon Health & Science University, Portland, OR USA

**Keywords:** Peritoneal dialysis, Tidal, Chronic kidney disease, Solute clearance

## Abstract

About 10% of all home peritoneal dialysis regimens in children with chronic kidney disease stage 5 are reported to involve some form of a tidal peritoneal dialysis (TPD) prescription. Despite this, there remain several gaps in how pediatric nephrologists approach the use of TPD. This stems from a combination of factors such as the confusing technical terminology pertaining to TPD, seemingly conflicting data on the risks, benefits, and indications for TPD, and lastly, limited published guidelines on the practical aspects of how to write a TPD prescription, based on the indication, in children. Our educational review, using evidence-based data, attempts to bridge this gap and provide an easy-to-use guide on the key practical aspects of TPD in children.

## Background

The 2021 United States Renal Data System annual data report indicates that 7.7% of the prevalent US population on dialysis received peritoneal dialysis (PD) as the primary dialysis therapy in 2019, a modest increase from 5.3% in 2009; in patients 0–17 years of age who received dialysis during 2019, 44% were started on PD at dialysis initiation [1]. Most pediatric patients receive their PD therapy with an automated bedside system on a nightly basis. Continuous cycling peritoneal dialysis (CCPD) refers to the PD modality that utilizes a machine to perform cycles or exchanges of dialysate during the night, mostly while the patient is sleeping. In CCPD, there are typically 6 to 8 exchanges nightly for 8–10 h; each cycle ends by trying to completely drain the peritoneal cavity of dialysate. Tidal peritoneal dialysis (TPD) is a form of CCPD in in which a volume of dialysate is maintained intraperitoneally between cycles rather than draining the peritoneal cavity fully with each cycle. In TPD, after the initial fill, only a portion of dialysate is drained for most cycles, maintaining more consistent contact between the peritoneal membrane and dialysate during each cycle. Subsequent partial drain volumes are replaced with new dialysate with each cycle. Periodically, the abdomen is completely drained to avoid abdominal distention, as will be discussed subsequently. TPD is designated with a tidal volume (TV), a tidal percentage, and a residual volume. TV is the amount of dialysate removed by each partial drain as part of the tidal prescription. Tidal percentage is the percentage of the total fill volume that the TV represents. Residual volume is the volume remaining in the peritoneal cavity with each TV exchange. For example, if a patient has a 2000-mL fill volume with a 60% tidal prescription, 1200 mL of fluid is exchanged during a tidal exchange; 1200 mL is the TV and 800 mL is the residual volume. The TV is managed by the automated machine, so it is expected to be accurate. The residual volume is variable, as there may be ultrafiltration (UF) (or absorption of the dialysate), and so technically, the true residual volume and tidal percentage may slightly vary from tidal cycle to tidal cycle.

TPD was originally developed, in part, to increase the contact of the peritoneal membrane with dialysate, and thereby increase solute clearance, based on animal studies first done in the 1960s and 1970s [2]. However, this has not been demonstrated in human studies, and there has been a shift in the use of TPD to focus on alleviating drain pain in patients.

## Theoretical benefits of TPD

There are several reasons why TPD might afford a significant advantage compared to other forms of automated PD (APD). With TPD, the residual volume theoretically maintains continuous contact of dialysate with the peritoneal membrane, thus maximizing actual time when dialysis is occurring, improving solute clearance. In addition, dialysate outflow times in TPD are regulated differently compared to other forms of APD, as discussed below [3].

The outflow of drained fluid during PD is biphasic; after an initial fast phase with constant rapid outflow, a slow phase abruptly ensues. This transition or ‘break point’ usually occurs after around 5 min after a 2-L dwell and adds to the total time on dialysis, without contributing to any substantive clearance [4]. This is further compounded in instances when the dialysate outflow rate is even slower than usual, as in settings of catheter malfunction or in patients with adhesions in the peritoneum; in such cases, the transition point is reached even earlier leading to suboptimal solute clearance. Using a TPD prescription that uses a regulated drain time, in which the drain cycle is terminated before the transition point is reached (as opposed to a preset drain time in other forms of APD), may improve the efficacy of dialysis by avoiding the slow drain phase and the gaps between the drain process and the subsequent fill.

Another potential advantage of TPD is in settings where patients experience significant pain during the drain phase of CCPD. Mechanistically, the residual volume (recommended to be about 200 to 300 mL in adults) in the abdomen theoretically allows the dialysis catheter to remain ‘afloat,’ minimizing irritation of the peritoneal lining and visceral organs caused by abutment of the catheter tip against those structures in a dry abdomen. This has been demonstrated to be effective in children [5], in adults [6], during pregnancy [7], and in patients with peritoneal calcifications and hemoperitoneum [8, 9]. This indication for TPD appears to have become the predominant reason why TPD is currently being used in adults and children. Data from the Baxter® database reveals that tidal prescriptions accounted for 12–13% of all prescriptions in children, and that over time, from 1997 to 2003, there was a steady decrease in the number of cycles and also a steady increase in TV, which is what one would expect if the goal of TPD was to maximize patient comfort as opposed to increase solute clearance, since only a small amount of residual dialysate is needed to keep the catheter afloat. In 1997, 58% of prescriptions had a TV greater than 75%; this increased to 75% in 2000 and to 92% in 2003 [10]. This shift in the use of TPD to focus on patient comfort has found its use both in the general population as well as in some unique settings, as discussed below.

The keys to successful TPD, therefore, are to ensure that there is a sufficient residual volume, to reduce drain pain, and adequate mixing of dialysate to optimize solute clearance. This was supported by data showing higher solute clearances with TPD in animal studies compared to continuous ambulatory PD [2].

## Human studies on TPD and its effect on solute clearance

Human data are not supportive of the finding in animal studies that TPD inherently leads to higher solute clearances. While clearances in many instances are improved with TPD, this is a result of higher dialysate flow rates (from the frequent exchanges that are sometimes used in TPD). When dialysis duration and total volume are kept constant, TPD does not result in an improvement in clearances (as measured by creatinine clearances and Kt/V urea) compared to conventional APD, at least with dialysate volumes up to 24 L/day [11].

In 2000, Juergensen et al. [11] studied 10 stable adult cycling PD patients and placed them into two groups to compare different TPD prescriptions on solute clearance. The total dialysate volume and duration were kept constant. Group I patients received dialysis with 15 L of dialysate over 9.5 h; on separate days, patients received 10%, 25%, and 50% TPD and non-tidal APD. Group II patients received dialysis with 24 L of dialysate over 9.5 h; they received 25% and 50% non-tidal APD on separate days. Analyses demonstrated that 10% and 25% TPD resulted in lower values for Kt/V urea than 50% TPD and non-tidal APD (*p* < 0.05); Kt/V urea was no different between the 50% TPD and the non-tidal APD groups. Creatinine clearances were the same across all modalities and prescriptions. Similarly, Piraino et al. [12] found that clearances of urea, creatinine, and phosphate were no different with 50% TPD and non-tidal APD when dialysate volume and duration were kept constant.

Balaskas et al. compared the effect of non-tidal APD with TPD in 12 patients, assessing differences in serum chemistries, blood pressure, body weight, and measures of patient well-being, including appetite and sleep. Again, using similar volumes of dialysis solutions per exchange and session and the same duration of dialysis, no differences were seen between the 2 groups in any of the studied parameters [13]. A more recent multicenter study further confirmed that TPD did not enhance clearances compared to CCPD. In this study, patients received CCPD and 25% and 50% TPD, each for 2 months, while keeping the dialysis volume and times constant. In this study, CCPD resulted in higher urea clearances compared to TPD. However, CCPD and 50% TPD resulted in comparable creatinine clearances, which were greater than with 25% TPD [14]. Vychytil et al. also noted that TPD did not improve small or middle molecule clearances when compared with non-tidal APD and further noted that patients with low and low average transport characteristics had lower clearances of urea and creatinine with TPD compared to non-tidal APD [15].

Possible explanations for why TPD, per se, does not improve clearances, despite its theoretical benefits, include incomplete mixing of the residual volume with fresh dialysate and/or the creation of stagnant fluid layers in the peritoneum. This inadequate mixing of the tidal with the residual volumes may be the result of the types of dialysate inflow in different dialysis modalities. TPD with a pulsatile inflow, as opposed to a gravity-dependent inflow, increases mixing and may result in better clearances. However, there are currently no commercially available cyclers with pulsatile flow, to make this a reality.

Nevertheless, the benefit that TPD continues to afford is that it may allow greater options in modifying different aspects of the PD prescription, such as the fill volume, the percent TV, and the frequency of cycles, without necessarily prolonging the total time that a patient has to remain on dialysis.

## Unique role of TPD in pediatric patients in the current era

Many small studies have been published on the use of TPD in children, who may have more to benefit from a tidal prescription, both for comfort, and to maintain solute clearance and ultrafiltration without increasing time on dialysis inordinately such that quality of life for the patient and family are adversely affected. The use of TPD in infants [16] and older children [17] may allow shorter dialysis treatment duration, at the expense of a higher dialysate flow rate resulting from a greater number of exchanges and shorter dwell times with TPD.

Hibino et al. [18] evaluated the efficiency of solute clearance and fluid removal in 17 pediatric patients who were transitioned to ‘large-dose TPD’ from non-tidal APD. Patients on non-tidal APD received a fill volume of 50 mL/kg or 1100 mL/m^2^ (maximum 2000 mL) and four to five exchanges for 8 to 10 h per night. The transition to TPD involved the same fill volume and dialysis duration, but with many more cycles (28 to 35) with a TV that was 50% of the fill volume; to prevent overfilling, patients underwent complete emptying of the abdomen every 6–7 tidal exchanges. The total dialysate volume in TPD, as expected, was about four times higher; it resulted in statistically significant increases (1.5 times higher) in the Kt/V urea. In the 9 patients who used a dialysate with the same glucose concentration for both dialysis prescriptions, the nightly fluid removal amount per hour with TPD was two times higher (*p* = 0.0039). Both the clearance of β2-microglobulin and serum β2-microglobulin were not significantly different, though the clearance of creatinine and phosphorus achieved with TPD improved. Based on their experience, the investigators proposed the use of a TV of 50% as being ideal, when increases in solute clearance are desired and large-volume TPD is being considered. While alternatives, such as increasing the number of cycles with standard APD prescriptions or the use of icodextrin for the day dwell, can be viable alternatives to TPD, especially in children with a high peritoneal membrane transport status [19], both may entail prolonging the total duration of dialysis and/or pose inconvenience to children during their school time. A dialysate flow rate exceeding 50 mL/kg/h has been suggested when TPD is being used to increase clearances in children [20]. In a small study of 6 children and their caregivers, children who were on CCPD were transitioned to TPD. The abdomen was initially filled with 35–45 mL/kg, and two to three tidal exchanges of 15–20 mL/kg were performed hourly. Daytime dwell volumes were reduced to 50–300 mL and the entire tidal session lasted 8 h. TPD shortened dialysis time (time savings of between 40 and 160 min per night) and eliminated daytime exchanges without sacrificing dialysis clearances. This sometimes required dialysate flows of 50–70 mL/kg/h atop a reserve volume of 20 mL/kg [20]. Within 6 weeks of training, all patients and families felt comfortable with this TPD prescription and they maintained proficiency through the 6 months of the study. Patients preferred the shorter dialysis time, and all asked to remain on TPD, since it was perceived to have improved the quality of life for these children and their families by decreasing the amount of time on dialysis.

Family perceptions were also explored in one of the largest prospective pediatric studies on TPD [5]. Seventeen children were randomized to a CCPD prescription with a fill volume of 1000 mL/m^2^ and 8 to 10 exchanges (resulting in a dialysate flow rate of 35 mL/kg/h) or to TPD with the same fill volume but many more exchanges (22–24 tidal exchanges with a 50% TV, resulting in an expected higher dialysate flow rate and total dialysate volume). At the end of the 6-month study period, the investigators in this study found that patients and parents felt safe with TPD (within a few weeks of starting TPD) and no patients reported dialysis-induced pain during TPD compared to three patients (23%) during CCPD. The total dialysis time was somewhat shorter on TPD, but this was not significantly different. In this study, Kt/V urea and creatinine clearance were higher during TPD in patients with high peritoneal membrane permeability, but similar in other patients; albumin and phosphate losses were comparable between the two groups.

In summary, while TPD is not necessarily a more effective modality in all children, it allows higher flow rates/volumes in shorter times and so may be of benefit in settings when suboptimal clearances and UF rates are noted such as in infants and older children who are high transporters. It may also be of value in patients with mechanical outflow problems in whom a larger fraction of the time on dialysis may get ‘wasted’ due to prolonged outflow times.

## Other settings when TPD may be of benefit

Other indications for TPD include patients with ascites, in whom a tightly controlled dialysate outflow is preferable [21], and those with peritoneal adhesions or malfunctioning catheters in whom the transition point is reached earlier such that the drain time can become inordinately prolonged leading to ineffective solute clearance, and lead to frequent machine alarms from slow and incomplete drainage [21].

Additionally, TPD may have a valuable role in the management of patients with acute kidney injury (AKI), especially when high dialysate flow rates are used. While data are limited, theoretically TPD may have a benefit in critically ill patients; since there is more consistent fluid in contact with the peritoneum, inflammatory mediators may get cleared more effectively. In practice, there is insufficient data from randomized controlled trials to definitely support this hypothesis [22]. In a small study in 2018, Al-Hwiesh et al. conducted a randomized trial of continuous venovenous hemodiafiltration (CVVHDF) versus continuous daily TPD in critically ill adults with AKI. Patients were randomized to receive either CVVHDF or TPD. In 62 CVVHDF patients, the replacement fluid rate was adjusted to keep the prescribed dialysis dose of 30 mL/kg per hour. In 63 TPD patients, TPD of 25 L/day was instituted with a fill volume of 2 L and a 70% TV. The 2 groups were comparable at baseline except that those patients randomized to CVVHDF had a higher percentage of volume overload. At 28 days, patients on CVVHDF experienced higher mortality compared with those on TPD (53% vs. 30%), greater infectious complications (17.7% vs. 9.5%), lower recovery of kidney function (35.5% vs. 60%), and a longer stay in the intensive care unit. On multivariate analysis, only sepsis (OR = 1.34, 95% CI = 1.16–1.64, *P* = 0.001) and dialysis modality (OR = 0.79, 95% CI = 0.68 to 0.11, *P* = 0.001) were associated significantly with mortality, with a lower odds of death with the use of TPD. This study, albeit small in study sample size, and involving a cohort that was not as sick as typically seen in critically ill patients with AKI, does provide an alternative to hemofiltration in patients with AKI in limited-resource settings and in instances where hemofiltration may not be feasible [23].

## Cautionary issues with the use of TPD

Among the things to consider with the use of TPD is cost. Since more dialysate bags are needed to perform large-dose TPD, costs invariably increase, too. Increasing the number of bags from 2 to 3, increases costs by 50% and increasing the number of bags from 2 to 4 doubles the cost for the dialysis fluids. Considering that the retail price of common PD solutions is approximately $50–75 per 6-L bag, this would increase the cost between US$18,250 and US$54,750 per year. If patients needed an extra manifold for their dialysis at night to place 5 or more bags, this would incur an additional cost. Also, in such a scenario, patients would require more time to set up and connect their PD circuit nightly, adding to their home care time. It would also require a larger storage area for supplies in their homes. In general, if prescribers are utilizing an alteration in prescription to use TPD to alleviate outflow symptoms during CCPD, the change in costs would be minimal, as the total volume of dialysis is usually similar to their original CCPD prescription.

Other potential issues with TPD are related to the need to predict and account for the volume that will be ultrafiltered during each cycle. It may be more difficult to predict initial UF precisely with TPD. If UF were excessive, there could be a negative impact on dialysis clearance from an excessive intraabdominal dialysate volume. It might also exacerbate existing hernias, create new abdominal wall defects, or cause a dialysis catheter fluid leak from the exit site. This is the rationale why early studies and long-term prescribing practices have led prescribers to completely drain the abdomen periodically during TPD sessions. However, most published studies on TPD have not demonstrated a higher incidence of such complications [18]. Furthermore, large-dose cyclic TPD was not associated with hypoalbuminemia or hyperglycemia in adult patients [18].

However, increased intraperitoneal volume (IIPV) events were seen more frequently in TPD and small fill volume therapies [24]. Specifically, they occurred when the total anticipated UF that was programmed was too low compared to the patient’s actual UF during tidal therapy. Overfilling events were also associated with patients bypassing the initial drain and in settings when drain volume alarms were set too low. IIPV events have been associated with abdominal or back pain, and leakage of dialysate into soft tissues including the pleural cavity causing dyspnea [25]. More serious IIPV events have included hydrothorax, and pericardial effusions [26–28]. Therefore, TPD should be used with caution in patients with pre-existing pulmonary or cardiac disease. On the other hand, if the actual UF is too low and lower than the programmed UF, the reserve volume will get depleted, and that may undermine the very purpose of TPD, which is predicated on having an adequate residual volume to continue to provide ongoing dialysis clearance. This is likely a main reason that patients with low or low average transporters do not have improved clearances with TPD.

## Practical considerations — the ‘how to’ of TPD

As emphasized in the preceding sections, abdominal pain during dialysis remains the main indication for TPD. Other less common indications for TPD are conditions in which higher solute clearances or UF are desired without increasing dialysis duration.

The ideal TPD prescription, therefore, is one that is targeted to the indication for TPD, and in general is one that would achieve the goal of pain- and alarm-free therapy with optimal clearances and UF, while delivering a safe treatment that avoids overfilling.

When contemplating a TPD prescription, the prescriber must decide on the following parameters, as mandated by the Baxter Home Choice® software (the most commonly used home cycler for pediatric patients in the USA), to ensure a safe and effective treatment:

1. The percentage of the initial fill volume that should be exchanged with each cycle (the TV) and amount that should stay in the peritoneal cavity (the residual volume);
2. Frequency of tidal full drain, i.e., how often to fully drain the peritoneal cavity; and
3. The net UF goal for the day.

There is no absolute recommended TV percentage necessary to keep the catheter afloat and decrease pain and drain alarms, and there is great variation in the literature on what has been used. In a cross-sectional survey of 6 adult dialysis units in Ontario, Canada, the TV percentage most frequently used varied between 75 and 80%; the most common indication for TPD in this cohort was drain pain [29]. As a general recommendation, the TV should be kept as high as possible (at least ≥ 50%), since only a small volume of residual dialysate is typically needed to keep the catheter afloat. This recommendation is aligned with the published pediatric experience with TPD, in which a TV of 50% was used and led to alarm- and pain-free dialysis treatments [5, 8, 20]. In a small subset of patients, such as those with prolonged drain times due to malfunctioning PD catheters, such an increase of TV may not be possible without compromising clearances; in such instances, a combination of high intraperitoneal fill volumes (e.g., initial fill volume 3 L in an adult-sized patient) combined with low TV (800–1000 mL) may allow dialysis to proceed without alarms [21].

In the few instances when TPD is considered for solute clearance, a TV of at least 50% has been proposed to achieve comparable clearances to standard APD prescriptions [11, 14]. This is in contrast to lower TVs (25%) which are associated with lower clearances as demonstrated in the previously referenced studies. To achieve higher clearances without prolonging dialysis duration, large-dose TPD can be considered, with a dialysate flow rate of at least 50 mL/kg/h [18, 20], using frequent exchanges (ranging from 20 to 35 per night) [5, 18] and a TV of 50%.

After determining the TV percentage, the prescriber must decide on the frequency of complete drainage of the peritoneal cavity. The main goal of complete drainage is to avoid overfilling (increased IIPV) and increased intraperitoneal pressure that can have grave consequences including death [29]. A commonly used practice is to completely drain the abdomen every three to four cycles. In the new model of the Baxter Home Choice® Cycler, the settings default to a complete drain every third cycle. This is a safety measure to prevent overfilling, but it can be modified by the prescriber. This may be desired and made less frequent in instances where patients are experiencing severe drain pain with each complete drain.

The final step is to enter the total anticipated (goal) UF for the therapy. This step is necessary to ensure that the volume that is ultrafiltered is removed along with the TV, and not retained until therapy is completed since that would lead to an overfilled state and its associated risks. The expected UF must be estimated carefully as adverse events may occur if the UF entered in the cycler program is set too low or too high. If the expected UF is set too low, progressive overfilling of the abdomen can occur from retention of the UF in the abdomen increasing the residual volume, which may lead to increased intraperitoneal pressure causing patient discomfort and as mentioned above, other serious complications. If the anticipated UF is set too high, the residual volume will be progressively depleted. This may lead to drain pain and would defeat the very purpose of initiating TPD. One way of estimating a patient’s UF is to review the previous actual UF achieved in the patient, the week prior to initiating TPD, and using 70% of that as the anticipated UF on TPD. Once tidal therapy is started, re-evaluating and re-assessing the actual UF is crucial to prevent overfilling or depleting the residual volume. In the new model of Baxter Home Choice® cycler, the minimum anticipated UF value defaults to 1000 mL per day. The prescriber can modify this number; the value entered has be greater than zero.

## Conclusions

In summary, while TPD has not been demonstrated in studies to inherently afford a benefit in solute clearance or UF compared to standard APD prescriptions, it does allow manipulation of the dialysis prescription to optimize dialysis, without inordinately prolonging the duration of dialysis. Such large-volume TPD can be considered in infants, high transporters, and in settings where the dialysis catheter is malfunctioning, and no other alternatives are available. The main role for TPD in today’s era is in the context of drain pain; by potentially floating the catheter in a residual volume, patient comfort and quality of life can be improved. There are limited data on whether TPD affords a survival benefit in critically ill patients with AKI. Lastly, close attention must be given to the TPD prescription — the choice of the TV, the frequency of complete drains, and the estimated UF can all impact patient safety and comfort, and affect outcomes.

**Key summary points**

- TPD entails always leaving a reservoir of fluid in the abdomen (residual volume) and refilling the abdomen with fresh dialysate (tidal volume) with each cycle
- Tidal prescriptions have their greatest utility in reducing drain pain by allowing the dialysis catheter to remain afloat in the residual volume and reduce irritation of the peritoneum
- On occasion, large-volume TPD can be used to optimize clearances and ultrafiltration when standard APD is not achieving desired outcomes
- Instances when large-volume TPD may be considered include infants, high transporters, and those with malfunctioning catheters
- A TPD prescription requires choosing and programming a tidal volume (as a % of the initial fill volume), the frequency of complete drains, and the net UF estimated to be achieved

## Multiple-choice questions

Answers can be found after the reference list

1. Which of the following is the most common indication for the use of TPD in the current era?
  A) Drain pain
  B) Abdominal hernias
  C) Low average transporters
  D) AKI from sepsis
2. The tidal volume refers to which of the following:
  A) The total nightly fill volume
  B) The volume of dialysate that is always left in the abdomen
  C) The volume of dialysate that is refilled into the abdomen after the first cycle
  D) The net UF expected with each cycle in TPD
3. Which of the following may be encountered as a complication of TPD if the expected UF is not accurately estimated?
  A) Overfilling of the abdomen
  B) Suboptimal clearance
  C) Lack of resolution of drain pain
  D) All of the above
4. Which of the following statements is true about TPD?
  A) Human studies have demonstrated an inherent benefit of TPD in improving solute clearance
  B) TPD is superior to CKRT in critically ill patients with AKI
  C) Patient comfort and machine alarms can be improved with a tidal prescription
  D) Ascites is a contraindication to the use of TPD
5. Which of the following is the true reason as to why programming periodic complete drains in a TPD prescription is important?
  A) To maximize solute removal
  B) To prevent overfilling of the abdomen
  C) To reduce drain pain
  D) To increase ultrafiltration
